# Comparison of the Current Situation of Equine Headshaking Syndrome in France and Switzerland Based on an Online Survey

**DOI:** 10.3390/ani12111393

**Published:** 2022-05-28

**Authors:** Laura Maxi Stange, Joachim Krieter, Irena Czycholl

**Affiliations:** 1 Institute of Animal Breeding and Husbandry, Kiel University, 24118 Kiel, Germany; jkrieter@tierzucht.uni-kiel.de (J.K.); iczycholl@tierzucht.uni-kiel.de (I.C.); 2 Pig Improvement Company (PIC), 100 Bluegrass Commons Blvd. Ste 2200, Hendersonville, TN 37075, USA

**Keywords:** horse, equine headshaking syndrome, therapies, welfare issue, survey study, signalment

## Abstract

**Simple Summary:**

Headshaking syndrome is a frustrating health problem in horses whose aetiopathogenesis is poorly understood. This study was dedicated to investigating signalment and therapeutic interventions in headshakers in France and Switzerland. To do this, an online survey was developed and distributed via the Internet. This showed that mainly middle-aged geldings were affected. The breeding distribution matched the breeding distribution of the relevant country. Furthermore, alternative healing methods were used by the horse owners, which have not yet been scientifically examined. Overall, it was possible to formulate conclusions regarding signalment and therapies used in practice.

**Abstract:**

Equine headshaking syndrome (EHS) is characterised as non-physical and involuntary movement of the horse’s head and neck. Although EHS is clinically simple to diagnose, its aetiopathogenesis often remains unclear. The aim of this study was to gain an overview of signalment and therapy possibilities used in France and Switzerland. To do this, an online survey was developed and distributed via newsletters. A total of 933 complete, answered surveys from France (*n* = 804) and Switzerland (*n* = 129) were evaluated. The median age in France was 12.4 years (CH = 14.3). Mostly geldings were affected (58.5%_FRA_, 57.4%_CH_). There was an association with Warmbloods in Switzerland (55.8%_CH_), but in France, in addition to Warmbloods (34.4%_FRA_), Thoroughbreds (27.2%_FRA_) were also affected. Moreover, horses affected by EHS often show stereotypical behaviour (15.7%_FRA_, 14.7%_CH_). A total of 38.4%_FRA_ and 67.4%_CH_ of horse owners utilised therapy measures, with nose covers being most commonly used (19.9%_FRA_, 30.2%_CH_). Horse owners resorted to alternative treatments that had not previously been studied in context with EHS (15%_FRA_, 20.9%_CH_). Conservative treatments, such as medication, were used by 5.4%_CH_ and 1.9%_FRA_. This study provides an overview of the status of horses affected by EHS in France and Switzerland and thus offers a fundamental step to understanding the consequences of welfare issues associated with EHS.

## 1. Introduction

Equine headshaking syndrome (EHS) has been a global problem for over 100 years and is still a problem in modern equestrianism. Despite a low prevalence of 1–1.5% for EHS, it appears as a cause of distress in horses and affects welfare [1]. EHS is considered pathological in the absence of any external stimulation [2,3,4,5]. It manifests itself as violent movements of the head and neck in horizontal, vertical, or rotational directions, as well as rapid vertical flips of the head [3,4]. Furthermore, the affected horses also snort, sneeze or rub their nose on their forelegs, the ground, or other objects [3,6]. The symptoms have been described in detail, documented, and divided into five degrees of severity by Newton et al. [7]. The evaluation system subdivides into mild clinical symptoms, moderate symptoms, limited rideability, and difficulty controlling to unrideable [7]. In addition, there are possible economic losses when selling headshakers due to a lack of tournament performances [8]. 

In general, headshaking is considered symptomatic when a primary disease is present [3,9]. The underlying diseases of symptomatic EHS were compiled by Cook [3] and have been systematically supplemented over the years by various researchers [4,10,11]. Disorders inducing EHS may be dental diseases, ear or eye infections, as well as disorders of the ligaments, e.g., in the horse’s neck. However, in 98% of cases, no physical cause can be found, and EHS is diagnosed as idiopathic [1,12]. Cases of EHS also appear characteristic of neuropathic pain. Usually, the fifth brain nerve (Nervus trigeminus) is affected, referred to as trigeminus-mediated EHS [12]. In this case, Nervus trigeminus is usually not impaired in its structure but rather in its function, which is caused by spontaneously occurring remission in some cases [1,4]. Various triggers (for example, wind, sun, dust) can lead to or exacerbate the specific symptoms of idiopathic as well as symptomatic EHS [4]. Many other circumstances besides neuropathic pain can trigger idiopathic EHS, such as stress or even riding style [1,3,4]. The poor understanding of the aetiopathogenesis also means that only a few scientifically proven and promising therapeutic methods are available. In particular, the use of medication such as Cyproheptadine and surgical interventions have been well studied to date [7,13,14,15]. Mills and Taylor [16] examined the effectiveness of nose nets in reducing the symptoms, with 70% relief in 30% of the cases [16]. However, owner surveys from English- and German-speaking countries show that other therapies are also used in practice. These include the use of alternative healing methods such as management changes, physiotherapy, or Traditional Chinese Medicine [17,18]. Additionally, geldings are overrepresented in those affected by EHS [4,13,17,18]. Furthermore, the English-speaking literature shows that Thoroughbreds are more likely to be affected by EHS, whereas Warmbloods are more likely to be affected in German-speaking countries [13,18].

Building on the survey study by Stange et al. [18] in Germany, this study intended to contribute to expanding the state of knowledge on the current situation of EHS in different European countries. Ross et al. [5] carried out a similar study to indicate the prevalence of headshaking within the UK.

The general aim was to provide an overview of horses affected by EHS in France and Switzerland, particularly with regard to breed distribution and the use of various therapies in practice.

## 2. Materials and Methods

### 2.1. Questionnaire Design

The target group of this questionnaire was horse owners of horses affected by EHS. Therefore, a questionnaire was designed with EFS Survey 18.4 Winter Releases^®^ (software by Questback) and divided into seven categories: introduction, signalment of headshaking horses, housing system and type of use, other diseases, symptoms as well as therapeutic measures. To ensure comparability, the questionnaire was adapted from Stange et al. [18] and translated into different European languages. The personal data of participants were treated confidentially and anonymised. In general, the questions were designed to be short and simple in order to avoid the possibility of participants failing to respond. The questionnaire contained 27 questions, subdivided into 7 open and 20 closed questions. The first question was a selection question before the introductory page, in which the language and the country of origin could be selected. Various types of questions were integrated to keep participation high. Predetermined answers were attached to the closed questions. In addition, the answer option, “don’t know”, was added to prevent termination of the survey. In general, comments were attached to each question to inform the participants of how to answer the questions. The answer fields were integrated for the open questions to be filled out in the participants’ own words. Furthermore, questions with multiple-choice answers were added.

All questions and associated answers, as well as the introduction page were included in the course of the evaluation presented below:

*Introduction:*

*Dear horse owners and riders!*

*Within the scope of a Ph.D. thesis at the Institute for Animal Breeding and Animal Husbandry at the Christian-Albrechts-University of Kiel, we want to gain information on the manifold causes of the equine headshaking syndrome.*

*A study on this topic has already been carried out in Germany in the form of a master thesis, and it will be a pleasure to send you the results by e-mail on request (contact me* via *lstange@tierzucht.uni-kiel.de). We would now like to extend this study to other European countries. For this purpose, we are looking for affected horse owners, riding associations, or trainers from other European countries to participate in our survey. Please fill out the following questionnaire if you own a horse, are affected by headshaking syndrome yourself, or know a horse (rider, trainer, or similar) to which you can refer. If you have/know more than one horse suffering from headshaking, please fill in the questionnaire once per horse. Participation in this study is voluntary and takes about 10 min.*

*Headshaking syndrome is characterised by spontaneous and uncontrollable displays of violent scenes of vertical or horizontal movements of the head and neck in horses, which differs from the normal movement pattern. Basically, the headshaking becomes pathological when the movement is performed without any recognisable external stimulus. In addition to vertical or horizontal movements of the head, various accompanying symptoms can occur. Although the symptomatology is known and described in detail, there is still a lack of generality about the aetiology and effectiveness of various therapeutic measures. By definition, symptomatic EHS is based on a primary disease (e.g., an injury of the cervical muscles), which is treatable, thus reducing the symptoms of EHS. When etiopathogenesis remains unclear, EHS is described as idiopathic. In some cases, it becomes dangerous to ride the horse.*

*In order to get an overview of your horse’s symptoms, we ask you to fill in the following questionnaire and, if necessary, give additional information in the fields provided. Please spontaneously state which answer appropriates the most to your horse. Of course, all information will be treated anonymously and will only serve as data basis for this Ph.D. thesis.*

*Thank you very much for your support!*

*Questions group 1: Information about your horse*

(1) *What gender is your horse? (Choose one answer.)*
*Closed question with three possible answers: mare, stallion, gelding.*
(2) *How old is your horse? (Indicate the age of your horse in years.)*
*Open question with answer field in which only numbers can be entered.*
(3) *To which type of breed does your horse belong? (Choose one answer.)*
*Closed question with five possible answers: Warmblood; Thoroughbred, Pony, Draft horse and others.*

*Questions Group 2: Information on housing system and type of use*

(1) *In which kind of stable do you keep your horse? (Choose one answer.)*
*Closed question with five possible answers: open stable (open stable: the simplest form of group housing with movement options), active stable (active stable: form of group husbandry with automated feeding and integrated incentives to move for horses), paddock box, box/daytime group housing, box housing, other (please specify).*
(2) *How do you use your horse? (Choose one answer.)*
*Closed question with five possible answers: sport horse with regular participation in tournaments, leisure sport with tournament ambitions, leisure horse, currently no use, permanently no use.*

*Questions Group 3: Information on other diseases*

(1) *Does your horse suffer from any other diseases besides headshaking? (Choose one or more answers.)*
*Closed question with six multiple-choice answers: healthy, respiratory diseases, eye diseases, gastrointestinal diseases, orthopedic diseases, other diseases.*
(2) *Do you observe stereotypies in your horse? Some examples of stereotypies: weaving, cropping, licking objects, scratching, walking in circles. (Choose one answer.)*
*Half-open questions with three possible answers and an attached comment field: yes, no, don’t know.*
*If yes, please enter the stereotype of your horse in the comment field.*

*Questions Group 4: Symptoms of headshaking syndrome in your horse*

(1) *Did a specific event cause the beginning of the headshaking? (Choose one answer.)*
*Half-open questions with three possible answers and an attached comment field: yes, no, don´t know.*
*If yes, briefly describe the event.*
(2) *How would you describe the symptoms of your horse? (Choose one or more answers.)*
*Closed question with seven multiple-choice answers: head up and down, frequent strong snorting, horizontal shaking of the head, rubbing the nose against the forelegs/floor/objects, reflex-like headbanging, reflex-like forehand strike, don’t know.*
(3) *When do you observe the symptoms? (Choose one answer.)*
*Closed question with five possible answers: at rest (box, pasture, paddock), in free run, on the lunge, at work (under the rider), don’t know.*
(4) *In which gait does the headshaking mainly occur? (Choose one answer.)*
*Closed question with four possible answers: walk, trot, gallop, don’t know.*
(5) *How often do you observe the headshaking? (Choose one answer.)*
*Closed question with four possible answers: <1 time per minute, 1–10 times per minute, >10 times per minute, don´t know.*
(6) *How would you rate the strength of the headshaking? (The intensity can be divided into five degrees of severity according to [7].*
*Grade 1: Intermittent and mild clinical signs; facial muscle twitching; rideable.*
*Grade 2: Moderate clinical signs; definable conditions under which they occur/develop; rideable with some difficulty.*
*Grade 3: Rideable but unpleasant ride; difficult to control.*
*Grade 4: Unrideable; uncontrollable.*
*Grade 5: Dangerous with bizarre behaviour patterns.*
*Choose the degree that suits your horse best.*
*Closed question with five possible answers: Grade 1, Grade 2, Grade 3, Grade 4, Grade 5.*
(7) *Does the headshaking occur seasonally? (Choose one answer.)*
*Half-open questions with three possible answers and an attached comment field: yes, no, don´t know.*
(8) *Did the symptoms change over time? (Choose one answer.)*
*Closed question with four possible answers: weaker, constant, stronger, don’t know.*

*Questions group 5: Therapeutic interventions*

(1) *Have you initiated therapeutic interventions against headshaking? (Choose one answer.)*
*Half-open answer with three answer options and a connected answer field: yes, no, don’t know.*
*If yes, what therapeutic intervention(s) did you use?*
(2) *Have you been able to achieve an improvement in symptoms by these measure(s)? (If yes, please choose one possible answer.)*
*Closed question with three possible answers: yes, no, don’t know.*

*Thank you very much for participating in the survey.*

### 2.2. Distribution of Online Survey

The participants were made aware of the survey via websites and articles in newsletters. With the help of the expertise of scientists from respective countries, the link to the questionnaire was distributed among horse owners and horse-related organisations. The introductory text contained the most important information on the process and content of the study. The survey link was publicly available to the participants for three months in 2021 in each country.

### 2.3. Statistical Analysis

The online survey software enabled the direct extraction of the raw survey data. The raw data were collected and processed externally. The evaluation was continued in the course of this study with the provided dataset. The first step was to analyse the raw data quantitatively. Statistical analysis was performed using SAS version 9.4 and Microsoft Excel 2016. To examine categorical relationships, cross-tabulations were prepared, and a Chi^2^-Test was carried out. PROC FREQ procedure was used for calculation. A significant association was assumed if *p* ≤ 0.05.

## 3. Results

### 3.1. Survey Findings

In France (*n*_FRA_ = 804) and Switzerland (*n*_CH_ = 129), 933 questionnaires were collected from participants in the online survey. The response rate to the questionnaire for France and Switzerland was 65.8%. The questionnaire was also sent to Italy (*n* = 13), Spain (*n* = 5), Russia (*n* = 0), Poland (*n* = 0) and Portugal (*n* = 7). However, the number of questionnaires returned was too small to evaluate for these countries. In the course of the evaluation (Section 3.3), trigger factors were categorised. Pollen and insects were combined under the term “allergies”. For example, stable or management changes that were the cause of EHS, based on the owners’ statements, are summarised under the item “posture”. “Diseases or trauma” describes, e.g., problems with the teeth or blunt injuries on the horse’s head.

### 3.2. General Description of Affected Individuals

The average age of all the participating horses in France was 12.4 years (range 3–30 years). The mean age of all horses which took part in the survey in Switzerland was 14.3 years (range 4–31 years). Within the sample from France and Switzerland, the gender distribution was reflected in a higher incidence of EHS in geldings (58.5%_FRA_; 57.4%_CH_). Table 1 shows the signalment of all horses affected by EHS. The results demonstrate that in Switzerland, more than half of the participating horses belonged to the type of Warmblood (55.8%_CH_). In France, the breeding type was distributed between Warmbloods (34.4%_FRA_) and Thoroughbreds (27.2%_FRA_). The category “Other breed” includes breeds with a special breeding goal (e.g., gait horses) or Crossbreeds. In France, mainly Western and Spanish horses were included. In Switzerland, participants mentioned Crossbreeds in addition to Western horses and horses of the baroque type.

Among the French horse owners questioned, 20.5%_FRA_ were sport-oriented tournament-riders, 30.7%_FRA_ were ambitious leisure riders, and 37.6%_FRA_ were leisure riders not participating in tournaments. 8.1%_FRA_ did not ride their horses for a short period due to recovery, and a further 3.1%_FRA_ of participants reported having their horses retired permanently. In Switzerland, 23.3%_CH_ were tournament riders, 27.1%_CH_ were leisure riders who occasionally attended competitions, and 39.5%_CH_ were purely leisure riders. 6.2%_CH_ were not riding their horses at the time of data collection, and 3.8%_CH_ of the horses were permanently retired. Based on the statements of the participants in the surveys, 41.2%_FRA_ of the French horses were affected by other diseases in addition to EHS (49.3%_CH_). Diseases were reported that affect the gastrointestinal tract, respiratory tract, eyes, teeth, and orthopaedic conditions. A significant connection between diseases of the gastrointestinal tract and stereotypes could be found within the French dataset (*p* ≤ 0.037; ɸ = 0.091). Table 2 details additional diseases of the horses affected by EHS.

In France, diseases such as skin problems (e.g., sarcoid, melanoma), Lyme disease, leptospirosis, and various metabolic diseases (e.g., equine metabolic syndrome, laminitis) were mentioned. In Switzerland, horses also occasionally suffered from “other diseases” such as skin problems, metabolic disorders (equine metabolic syndrome), or dental problems. In addition to EHS, 15.7%_FRA_ of the horses in France showed stereotypical behaviour, such as weaving, cribbing, or running in circles. In Switzerland, 14.7%_CH_ of the horses showed stereotypes.

### 3.3. Symptoms and Therapies

In 22.3%_FRA_ (23.3%_CH_) of the cases, respondents indicated that the EHS had been preceded by a special event. This question was rejected by 45.3%_FRA_ (46.5%_CH_) of the participants. Causes for the manifestation of EHS were mentioned by 25.7%_FRA_ (27.0%_CH_) of the owners. These may be allergies (4.1%_FRA_, 0.8%_CH_), stress (0.6%_FRA_, 1.5%_CH_), training (4.7%_FRA_, 4.6%_CH_), diseases or trauma (5.8%_FRA_, 7%_CH_), type of husbandry (4.2%_FRA_, 7%_CH_), vaccination (1.3%_FRA_, 3.8%_CH_), season or weather influences (3.4%_FRA_, 1.5%_CH_), equipment (0.2%_FRA_, 0.8%_CH_). Horses from all countries mostly show vertical head movements as a symptom of EHS; the symptoms were most frequently observed while being ridden across all countries. Table 3 shows the frequencies in percent for the type of symptoms of EHS in all affected horses in France and Switzerland. Furthermore, it presents the situation in which the EHS is mainly observed, as well as the gait and frequency in which EHS occurs.

Data from French participants also showed that the symptoms of nose rubbing and vertical shaking (*p* ≤ 0.001; ɸ = 0.114), as well as snorting, were connected (*p* ≤ 0.001; ɸ = 0.259). In Switzerland, snorting was associated with vertical movements (*p* ≤ 0.030; ɸ = 0.191) and forehand strokes (*p* ≤ 0.01; ɸ = 0.226). Rubbing the nose showed a significant connection with heavy head flicks in the Swiss data set (*p* ≤ 0.001; ɸ = 0.317) as well as forehand strokes (*p* ≤ 0.002; ɸ = 0.272). The Swiss data shows that an association between the horizontal direction of movement and the presence of stereotypes could be proven (*p* ≤ 0.027; ɸ = 0.237), and in France, snorting is connected with stereotypes (*p* ≤ 0.010; ɸ = 0.107). Furthermore, more than half of all affected horses in all countries showed symptoms of EHS with a frequency of 1–10 x/min based on the owners’ responses (52.1%_FRA_, 58.1%_CH_). Based on the scale of Newton et al. [6], participants were asked for the severity of the symptoms. The distribution within the sample for the French participants was 26.4% grade 1; 36.6% grade 2; 29.6% grade 3; 6.2% grade 4 and 1.2% grade 5. There was a correlation between the severity of symptoms and the kind of symptom (vertical EHS) within the French data set (*p* ≤ 0.001; ɸ = 0.312). 21.7% of the affected horses in Switzerland were allocated to grade 1; 43.4% grade 2; 28.7% grade 3; 4.6 grade 4, and 1.5% grade 5. The Swiss data set showed a correlation between the strength of EHS and with use of a therapy (*p* ≤ 0.025; ɸ = 0.369). In addition, the survey data show that a seasonal dependence of EHS symptomatology can be observed in 60.7%_FRA_, and 66.7%_CH_ of the affected horses. About a quarter of the participants (26.9%_FRA_, 25.6%_CH_) answered in the negative, and 12.4%_FRA_ and 7.7%_CH_ gave ‘don’t know’ as an answer. Various statements were made by the participants in this study on the development of EHS, including a seasonal influence. Furthermore, vaccination (1.3%_FRA_, 4.6%_CH_) as well as stress (0.5%_FRA_, 1.5%_CH_), training status or the influence of the rider (4.7%_FRA_, 4.7%_CH_) are mentioned by the participants as well as the housing conditions (4.4%_FRA_, 7%_CH_) and trauma and diseases that are not described in detail (4.6%_FRA_, 7%_CH_).

The French horse owners did not choose any therapy in 61.6%_FRA_ of the cases. In contrast, 67.4%_CH_ of the participants in Switzerland used therapeutic options to improve EHS symptoms. The therapeutic measures used have been summarised based on the participants’ free statements. The most common tool used to reduce symptomatology was the nose net (19.9%_FRA_; 30.2%_CH_). In France, 8%_FRA_ of the horses received osteopathy as a treatment for symptoms, and another 7%_FRA_ of the owners stated that they treated the symptoms with alternative healing methods. These include, e.g., acupuncture, Traditional Chinese Medicine, or homeopathy. A change in feeding or the administration of additional feeds as well as a change in husbandry conditions was attempted by 5%_FRA_ or 1.1%_FRA_ of the participants. A change in riding style and equipment was described by 1%_FRA_ and 0.7%_FRA_ of horse owners. Conservative treatment measures were also used: 0.5%_FRA_ participants reported an operation on the trigeminal nerve as treatment. Another 1.9%_FRA_ used medication (12 Cortisone, 1 Cyproheptadine, 2 unknown medications). Percutaneous electrical nerve stimulation was used by 0.2%_FRA_ of the participants. Furthermore, a significant connection between rubbing (*p* ≤ 0.001; ɸ = 0.243) and snorting (*p* ≤ 0.001; ɸ = 0.148) symptoms and the application of therapy could be found in the French dataset. The Swiss participants stated that they had chosen alternative healing methods as therapy (8.5%_CH_). 12.4%_CH_ of the participants used osteopathy or physiotherapy.

Furthermore, changes in posture (3.1%_CH_), feeding (1.5%_CH_), equipment (2.3%_CH_), or riding style (7%_CH_) were described as therapeutic measures. There was no surgical intervention. 5.4%_CH_ horse owners administered medication, and one participant reported using percutaneous electrical nerve stimulation. An association between rubbing (*p* ≤ 0.015, ɸ = 0.255) and snorting (*p* ≤ 0.041, ɸ = 0.223) and the use of therapy could be proven for the Swiss dataset. In the survey in France, the owners stated that an improvement could be observed in 27.1%_FRA_ of the horses. In Switzerland, 54.3%_CH_ of the owners surveyed described a reduction in symptoms. The question was answered in the negative by 26.5%_FRA_, 13.2%_CH,_ and a further 26.1%_FRA_, 11.6%_CH_ were not sure whether an improvement could be observed. 20.3%_FRA_, 20.9%_CH_ of the participants skipped this question during the course of the questionnaire.

## 4. Discussion

This study on EHS at the European level builds on the study on German horses affected by EHS by Stange et al. [8,18]. In the course of this study, the questionnaire was tested for validity and reliability with the help of experts in the field [18]. Data collection with the use of an online survey is a suitable tool for obtaining large samples [19]. The software EFS Survey 18.4 Winter Release^®^ enables large numbers of participants and comprehensive data collection, which is why it was used in this study as well. The owner survey in other European countries (e.g., Italy and Spain) only achieved small sample sizes, which can be explained by the fact that the link to the questionnaire did not reach sufficient numbers of horse owners due to insufficient presence on the Internet. 

In France and Switzerland, the link was spread via popular horse-related magazines, and we were able to collect 933 questionnaires from French and Swiss horse owners. The response rate for the French/Swiss survey with 65.6% is higher than that of other studies (39%, 42%, 59.5%) [13,17,18]. The disadvantage of an online survey is that participants may fill out the questionnaire improperly and thus provide incorrect information [19]. Due to this limitation and because this dataset refers to personal statements from horse owners, responses should be considered with caution. In addition, it could not be tested whether the participating owners can distinguish idiopathic headshaking from physiological head movements. Nevertheless, this type of data collection proved to be a useful tool in obtaining a large sample and information about horses affected by EHS in different countries. However, it remains unclear how many of the affected horses suffer from symptomatic or idiopathic EHS.

Based on previous studies, the median age of horses affected by EHS is between eight and twelve years [4,5,13,18]. This could be confirmed with the help of this study. Based on this study, no statement about the age of onset of EHS can be made. Likewise consistent with the literature, this study shows that EHS mainly affects geldings (70%, 62.5%, 71.5%, 64.4%) [4,13,17,18]. Ross et al. [5] could not identify a significant connection to gender. In this study, 58.5%_FRA_ of the horses in France were geldings and 57.4%_CH_ in Switzerland. An explanation for the frequent occurrence in geldings is still missing. For instance, treatment with gonadotropin-releasing hormone in affected geldings has shown no improvement in symptoms [20].

In Switzerland, Warmbloods are primarily affected by EHS (55.8%_CH_). This overrepresentation is also stated in Germany, where 55.4% of the horses belong to the Warmblood type [18]. On the other hand, the data from France show that many Thoroughbreds are affected (27.2%_FRA_). This is consistent with the English-speaking literature, which assumes a prevalence of high-blooded horses [13,17]. In contrast, Ross et al. [5] found no evidence of breed with EHS within the horse population in the UK. However, in France, Thoroughbreds tend to be bred rather than Warmbloods [21], which may explain the higher proportion of Thoroughbreds within this study. Hence, it can be assumed that the breeding distribution within the group of horses affected by EHS corresponds more to the breeding distribution of the respective country, which results from the country-specific breeding history.

The survey reported that almost half of the horses in Switzerland suffer from other diseases (49.3%_CH_) in addition to EHS. In France, other diseases affected 41.2%_FRA_ of the participating horses. The horses were mainly affected by gastrointestinal diseases (8%_FRA_; 11.5%_CH_) or suffered from other diseases affecting the skin or metabolism (16.6%_FRA_; 10.1%_CH_). In particular, the frequent occurrence of stereotypes in all countries surveyed should be emphasised (15.7%_FRA_; 14.7%_CH_). Stange et al. [18] also found this increased occurrence in 18.7% of the horses surveyed in Germany. Stereotypes usually arise due to problems in husbandry conditions [22]. Within the scope of this study, no connection could be found between EHS stereotypes and housing conditions. However, 3.6% of the French horse owners cited husbandry as a cause of EHS. In a survey of horse owners in Switzerland, it was found that 3.5% of the horses (*n* = 2341) exhibited stereotypes [23]. Other studies, e.g., from Italy, assume a prevalence of 2.5% [24]. Therefore, the number of stereotypes within this group seems particularly high.

Furthermore, this study demonstrates a significant correlation between horizontal headshaking and stereotypical behaviour. An explanation might be that the horses suffer from stereotypes and physical symptoms such as EHS occur because of reduced welfare. On the other hand, horses suffer from EHS, and stereotypes develop as a result. However, due to the selected study design, it cannot be determined whether EHS or stereotypical behaviour appeared first. Actually, horses with EHS seem to have compromised welfare. Thus, this interaction should be investigated more closely in further studies.

From the current literature, it is known that symptoms are usually more pronounced while being ridden and in higher gaits [7,13,25]. Even in this study, the majority of the horses (65.4%_FRA_, 66.7%_CH_) showed EHS symptoms mainly while being ridden. However, up to a quarter of the horses (24.9%_FRA_; 16.3%_CH_) showed increased symptoms of EHS even at rest. Recent studies have indicated that EHS symptoms increase over time [1,25,26] and may explain why horse owners observe the symptoms under resting conditions. Nodding and, more rarely, shaking movements are known from the literature, with the vertical direction of movement occurring more frequently, and the occurrence of various accompanying symptoms such as snorting and rubbing is mentioned [3,4,6,27]. Vertical headshaking is the most common direction of head movement mentioned in this owner survey (71.8%_FRA_, 75.2%_CH_). Furthermore, nose rubbing as a symptom often occurs together with other symptoms, which can be shown in this study based on the correlations found. The typical EHS movements are often paired with the accompanying symptoms [3,6]. This is reflected in the course of this study. Using the classification of Newton et al. [7], the horses were divided into degrees of severity by their owners. 30%_FRA_ of the French and almost 29%_CH_ of the Swiss participants classified their horses as “difficult to control”. In addition, 7.5%_FRA_ (6.1%_CH_) of the horses were classified as “unrideable, uncontrollable” or “dangerous”. It remains undisputed that symptoms of EHS can lead to dangerous situations when riding and handling affected horses [4,13]. A seasonal dependency with an increased occurrence of symptoms in the summer months is mentioned in the current literature [28] and can be underlined by the results of this study. The participants of this study named various causes, such as vaccinations for the occurrence of EHS. This has not been adequately researched scientifically yet. However, Aleman et al. [29] determined the presence of EHV-1 in the trigeminal ganglia of horses with idiopathic headshaking but could only detect it in one of eight headshaking subjects and with that ruled out EHV-1 infection as a cause. The influence of other vaccinations is not yet known. The participants also list stress, training condition, or the rider’s influence, as well as the husbandry conditions. So far, there is no scientific evidence for this, but these causes are mentioned in the literature [9,30]. It should be added that it is difficult to obtain reliable information on such questions due to the study design chosen. Nevertheless, it gives a good impression of the current situation in the population and further need for research.

The development of a suitable therapy is therefore of great importance. In addition to surgical procedures, medication can be used to reduce EHS. The horse owners mainly indicated medication administration such as Cortisone, probably in connection with allergic events. Cyproheptadine is an antihistamine and has been studied in the treatment of EHS by Madigan et al. [31] but without success and was only used once by the surveyed horse owners. However, side effects are to be expected, and the financial outlay is relatively high for both medication and surgery, which probably means that these therapies are rarely administered.

Nevertheless, in France, the level of insurance for horses is low, especially for horses under the age of 15 years [32]. Based on this, it can be explained that in France, fewer horse owners seek veterinary intervention. The nose net is most frequently used as a therapy (19.9%_FRA_, 30.2%_CH_). The mechanism of underlying symptom reduction through a nose net has not yet been elucidated. However, a study by Mils and Taylor [16] has shown that the use of a nose net leads to a reduction of symptoms of up to 70% in 30% of all participating horses. It is important to emphasise that using a nose net does not treat the cause rather than the symptoms. The nose net is probably used frequently because it is cheap and helpful. 

In France and Switzerland, horse owners resorted to alternative treatments that have not previously been studied in connection with EHS (15%_FRA_; 20.9%_CH_). These include acupuncture, physiotherapy, osteopathy, and Traditional Chinese Medicine. Moreover, horse owners report an improvement or lack of symptoms due to a change in riding, equipment, or husbandry conditions. However, the use of therapy and the absence or reduction of symptoms is still no indication of a causal connection. The symptoms improved in half of all horses in Switzerland and a quarter of the horses in France. Due to the chosen methodology, it is unclear which therapy contributes the greatest success to symptom reduction.

Nevertheless, it is clear that the use of therapy contributes to an improvement in symptoms. EHS is difficult for veterinarians to assess because it can vary greatly between the animals and is unpredictable [16]. The study situation is still limited, and this is why collecting data from owners can be seen as a suitable tool, especially with regard to therapeutic interventions.

Building on the German study by Stange et al. [18], this study aimed to gain an overview of horses affected by EHS in European countries and reach larger samples, which was successful, especially in Switzerland and France due to a good presence in popular scientific journals. In general, it was found that the breeding distribution within the group of horses affected by EHS matches the breeding distribution of the specific country. Overall, horses with EHS are limited in their welfare. Nevertheless, a secure diagnosis is essential to initiate successful therapy. Above all, alternative therapies should be investigated in further studies.

## 5. Conclusions

This work contributes to the knowledge about EHS and provides an overview of the therapies used in practice. Knowledge of the global prevalence of EHS is fundamental for a better understanding of the syndrome and gaining an overview of possible effective treatment options. In order to gain expertise, the aim should be to conduct extensive research in other countries to make comparisons possible and to gain a better understanding of the aetiopathogenesis of EHS.

## Figures and Tables

**Table 1:** Median age, gender, and breeding distribution of all participating horses for each country are given in percentages.

Variable	Variable Details	France (*n* = 804)	Switzerland (*n* = 129)
Median age		12.4	14.3
Gender distribution	Mare	39.7	41.1
	Gelding	58.5	57.4
	Stallion	1.9	1.5
Breeding distribution	Warmblood	34.4	55.8
	Thoroughbred	27.2	11.6
	Draft horse	11.4	13.1
	Pony	18.5	10.1
	Other breed	8.0	9.3

**Table 2:** Diseases besides headshaking in all participating horses given in percentages.

Diseases	France (*n* = 804)	Switzerland (*n* = 129)
Gastrointestinal diseases	8.0	11.5
Respiratory diseases	7.4	10.8
Eye diseases	4.8	6.1
Orthopedic diseases	4.4	10.8
Other diseases	16.6	10.1

**Table 3:** Behavioural characteristics of equine headshaking syndrome (EHS) given in percent (direction of movement, situation, gait, and frequency).

Variable	Behavioural Characteristics	France (*n* = 804)	Switzerland (*n* = 129)
Symptoms of EHS/Direction of movement	Vertical	71.8	75.2
	Horizontal	20.8	24.0
	Rubbing the nose	62.9	62.8
	Snorting	25.9	31.0
	Forehand stroke	17.8	23.3
	Heavy headshaking	58.7	63.6
Situation	Rest	24.9	16.3
	Free movement	2.1	3.9
	On the lunge	5.6	3.5
	While being ridden	65.4	66.7
Gait	Walk	37.7	41.9
	Trot	40.7	36.4
	Canter	12.8	3.1
Frequency	<1 x/min	24.0	17.8
	1–10 x/min	52.1	58.1
	>10 x/min	15.2	12.4

## Data Availability

Data available on request from the corresponding author.
